# Processing of Factor XII during Inflammatory Reactions

**DOI:** 10.3389/fmed.2016.00052

**Published:** 2016-11-04

**Authors:** Bernard Nico Jukema, Steven de Maat, Coen Maas

**Affiliations:** ^1^Department of Clinical Chemistry and Hematology, University Medical Center Utrecht, Utrecht, Netherlands

**Keywords:** factor XII, inflammation, bradykinin, plasmin, elastase

## Abstract

The contact system was originally identified as an obsolete part of the coagulation system, but it has been repeatedly implicated in inflammatory states, such as infection, as well as in allergic- and chronic inflammatory disease. Under these conditions, there is surprisingly little evidence that factor XII (FXII) acts as a coagulation factor, and its activity appears to be mainly directed toward activation of the kallikrein–kinin system. The contact system factors interact with pathogens as well as cells of the (innate) immune system on several levels. Among others, these cells may provide negatively charged surfaces that contribute to contact activation as well as release enzymes that feed into this system. Furthermore, cellular receptors have been identified that bind contact factors at sites of inflammation. Based on the accumulated evidence, we propose a model for enzymatic crosstalk between inflammatory cells and the plasma contact system. During these reactions, FXII is enzymatically cleaved by non-contact system enzymes. This generates unactivated FXII fragments that can subsequently be rapidly activated in the fluid phase. The resulting enzyme lacks procoagulant properties, but retains its pro-inflammatory characteristic as a prekallikrein activator.

## Introduction

Continuous maintenance of vascular integrity is essential for effective blood circulation and overall survival. When tissues are infected or injured, local increases in vascular permeability and controlled inflammation are needed for protection and repair. The short-lived peptide bradykinin is a well-known mediator of inflammation and vascular leakage. To this day, the plasma contact system is considered to be the most important source of intravascular bradykinin production. However, the physiological mechanisms that drive this system to generate bradykinin remain elusive.

The plasma contact system consists of the two proteases, factor XII (FXII) and plasma prekallikrein (pro-pKal), and their non-enzymatic cofactor high molecular weight kininogen (HK). These coagulation factors spontaneously activate in the presence of negatively charged surfaces, which [as previously reviewed in Ref. (1, 2)] can be non-natural (e.g., kaolin) or cell-derived (e.g., polyphosphate). Surface-binding of FXII is accompanied by a conformational shift (3). This may generate the first spark of enzymatic activity (4). In chorus, HK (in complex with pro-pKal) also binds to the negatively charged surface, thereby presenting pro-pKal for activating cleavage by active FXII (FXIIa). In turn, activated plasma kallikrein (pKal) can reciprocally cleave and activate more FXII, forming a powerful activation feedback loop that can avoid inhibition by C1-esterase inhibitor (C1inh). In part, this can be attributed to the negative charge of the activating surfaces, which electrostatically repel C1inh (which is also negatively charged). When sufficient amounts of FXII activate on the surface, FXIIa activates factor XI (FXI) in a mechanism that closely resembles pro-pKal activation. Deficiencies in contact factors were originally identified as *in vitro* defects in surface-mediated clotting reactions (5, 6). As a direct result, it is generally thought that contact activation will inherently lead to blood coagulation. Mysteriously, deficiencies in the contact factors are without bleeding diatheses, providing reasons to believe that the contact system has become redundant for physiological hemostasis. But is activation of blood coagulation by the contact system truly its first and foremost important function?

**Figure 1:**
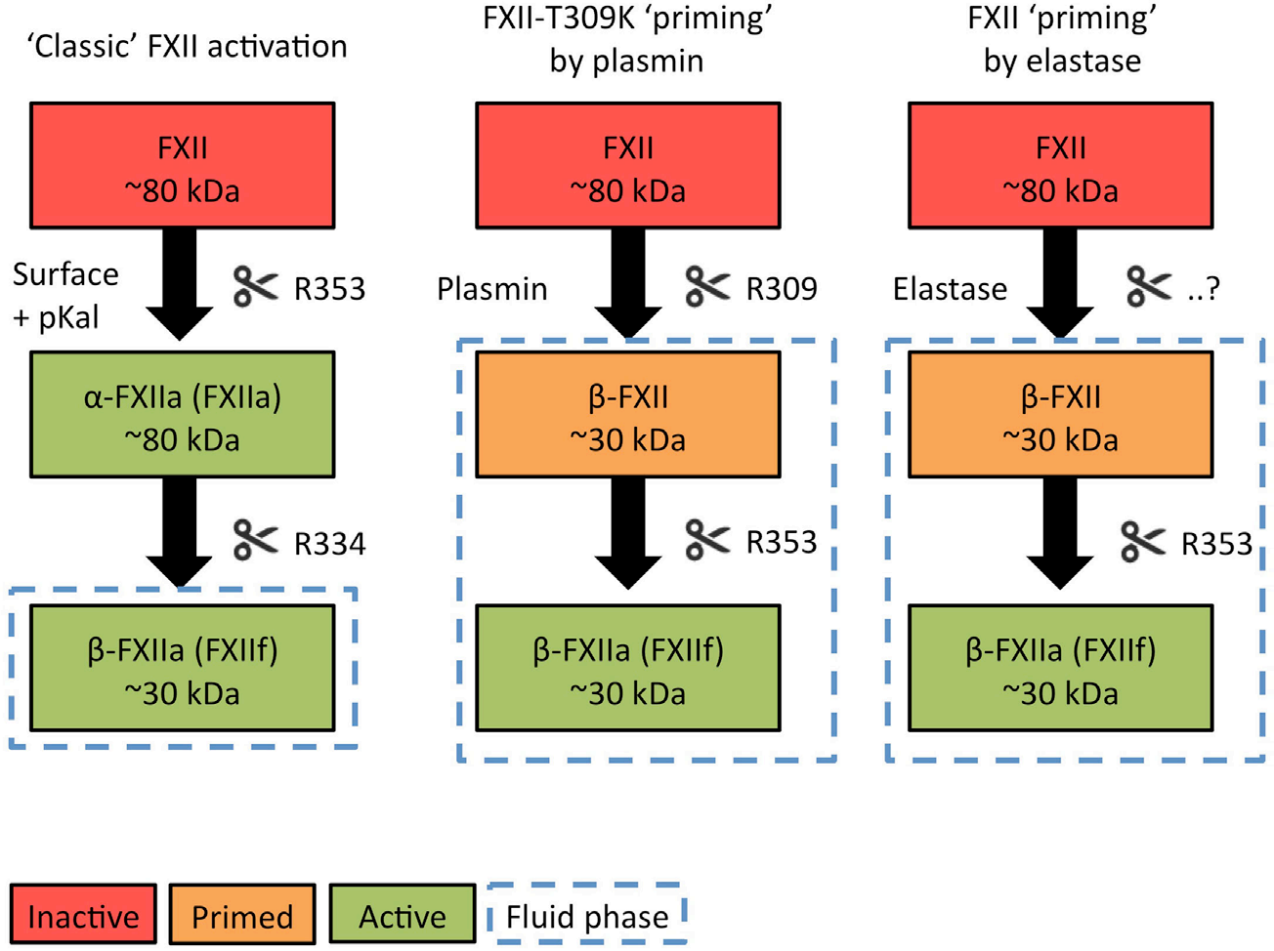
**A model for two-stage activation of Factor XII**.

At this point, it is noteworthy that only a subset of negatively charged activators of the contact system support activation of FXI by FXIIa. Generally, these surfaces are insoluble particles (7–9). However, a second type of contact system activator (generally negatively charged soluble polymers) is unable to support FXII-driven blood coagulation *in vitro* or activate FXI *in vivo* (8, 10). Surprisingly, this class of activators still powerfully promotes pKal activity and bradykinin production. The fundamental principles that make the contact system “decide” whether or not to trigger coagulation in response to specific activators are still unknown, but we propose that this is related to alternative conformational changes that FXII undergoes when it binds to activating surfaces (7). Furthermore, earlier biochemical investigations have pointed out that surface-bound FXII becomes activated in a step-wise mechanism (Figure 1, “Classic” contact activation). A first pKal-mediated cleavage activates FXII into a full-length two-chain molecule with surface-binding and procoagulant characteristics. Further cleavage by pKal fragments the molecule, allowing it to dissociate into solution. This enzymatic fragment has lost the ability to activate FXI, but can still act as a powerful pro-pKal activator (11).

## The Contact System in Inflammatory Pathology

The contact system has attracted strong scientific attention as a result of its contribution to pathological thrombus formation and the potential it holds for developing safe antithrombotic strategies without an associated bleeding risk (12). However, this system has also been repeatedly implicated in acute inflammatory and allergic reactions, as well as chronic inflammatory disease, often without a clear link to the coagulation system.

### Sepsis

Patients with sepsis undergo a systemic inflammatory response and can experience fever, hypotension, tachycardia, and organ failure (13). Sepsis can be caused by various pathogens, although bacterial infection is most common. When primates are challenged in an *Escherichia coli*-induced sepsis model, contact system activity is observed in the systemic circulation (14–16). Inhibition of contact system activity attenuates complement activation and diminishes neutrophil degranulation. Resultantly, overall survival rates increase. However, when FXII is inactivated by a blocking antibody, disseminated intravascular coagulation still occurs, indicating that the contact system is not responsible for the thrombotic aspect of this pathology. Presumably, expression of tissue factor by circulating cells and diffuse vascular damage, leading to subendothelial exposure, are the driving factor behind this prothrombotic aspect of sepsis. When guinea pigs are experimentally infected with *Pseudomonas aeruginosa* (*P. aeruginosa*), it triggers development of peritonitis (17). Activation of the contact system is observed during these infections and plays a role in this pathology: antibody-mediated depletion of FXII prevents the onset of septic shock. In contrast, depletion of α2-macroglobulin, which has the ability to inhibit pKal during these infections, exacerbated the outcome. While these two studies highlight a prominent role for bradykinin production in septic shock models, it is important to note that not all sepsis models show identical results. In a porcine sepsis model with *P. aeruginosa*, the onset of symptoms was not influenced by a kinin B2 receptor antagonist. However animals did recover faster as a result of this treatment (18), suggesting that the contact system does not play a lead role throughout the entire pathological process. In a similar porcine infection model with *Neisseria meningitides*, administration of a kinin B2 receptor antagonist had no effect (19). This raises the question whether all forms of sepsis are accompanied by contact system activation.

In a human study of systemic inflammatory response syndrome, continuous infusion of a bradykinin antagonist had no overall effect on the 28-day survival (20). However, a subset of patients with a Gram-negative bacterial infection did show improvements in recovery. Another study reports that levels FXIIa–C1inh and PK–C1inh complexes were transiently increased in 40% of patients during the course of their sepsis (21). These observations in human patients parallel observations in animal studies and suggest that not all types of infection that lead to sepsis act on the contact system in the same manner. Some pathogens trigger contact system activity *via* outer surface components: Curli-expressing *E. coli* have been shown to directly bind and activate the contact system on their surface (22). Furthermore, *E. coli, Bacteroides fragilis, Bacteroides vulgatus*, and *Fusobacterium mortiferum* LPS have been postulated to be able to directly activate FXII (23, 24). However, other pathogens appear to trigger contact system activity in an enzymatic manner. For example, *P. aeruginosa* expresses a form of elastase that, after administration in guinea pigs, provokes massive consumption of FXII, PPK, and HK and triggers bradykinin formation, recapitulating key features of pseudomonal sepsis (25). Several other microbial enzymes with similar functions have been identified (26). Three main groups of proteinases can be distinguished: (I) those that activate FXII, but not pro-pKal; (II) those that can activate both FXII and pro-pKal; and (III) those that directly liberate bradykinin from HK. Finally, recent studies have shown that bacterial strains that carry direct plasminogen activators (e.g., streptokinase) can trigger plasmin-triggered bradykinin production *via* the contact system (27), which is highly reminiscent of earlier studies that identified plasmin as an activating enzyme of FXII (28) as well as recent findings that implicate plasmin as FXII-activating enzyme in hereditary angioedema (HAE) (29). This may help to explain the changes in blood pressure that take place during sepsis but also possibly points toward a bradykinin-dependent mechanism of pathogen host invasion.

### Anaphylaxis

Anaphylaxis is a severe allergic reaction with a possible deadly outcome. Attacks can be triggered in reaction to food, insect bites and/or stings, and medication. As a result, patients can experience gastrointestinal and skin manifestations, as well as arrhythmias, bronchial constriction, and vascular leakage, which causes hypotension. These effects are generally thought to be mainly due to extensive degranulation of mast cells and basophils. However, several lines of evidence point to a role for bradykinin in the exacerbation of allergic reactions.

Patients who undergo attacks of anaphylaxis show strong consumption of contact system factors (30, 31). Interestingly, plasminogen activation is simultaneously seen in these same patients (32). Consumption of contact factors has also been reported in IgE-mediated mouse models for anaphylaxis (10). This raises the questions of how (and why) the contact system activates during anaphylaxis. Upon mast cell and basophil degranulation, a wide variety of vasoactive mediators and proinflammotory effectors are secreted which belong to a wide variety of cytokines, chemokines, or lipid mediators (33). Alongside these substances, the highly sulfated glycosaminoglycan heparin is also secreted. While heparin is mostly known for its anti-coagulant properties in the clinic, for mast cells, heparin acts as scaffold and carrier for several proteases and is essential for the proper morphology of the secretory granules (34, 35). To execute this function, heparin shares many properties with negatively charged polymers that can active the contact system. Indeed, when heparin is isolated from peritoneal mast cells and subsequently added to plasma, or administered intravenously to mice, contact system activation and bradykinin formation ensues (10). It should be remarked that therapeutic heparin preparations do not trigger significant or dangerous systemic contact system activation, unless highly charged impurities are present (36). However, when mast cells release heparin *in vivo*, significant contact activation follows. In IgE-dependent mouse models for anaphylaxis, genetic and pharmacological targeting of the contact pathway attenuates symptomatic hypotension and cutaneous swelling (10). As such, it is proposed that mast cell heparin is an important endogenous contact system activator in anaphylaxis in mice and humans. Furthermore, these experiments indicate that mast cell/histamine driven allergic reactions are mechanistically coupled to bradykinin production and do not present exclusively operating mechanisms for vascular leakage.

### Multiple Sclerosis

Multiple sclerosis (MS) is a severe neuroinflammatory disease, which affects local function of the central nervous system (CNS). Patients can experience loss of their vision, muscle coordination, and sensation. While the precise cause of MS is enigmatic, the general consensus is that MS is caused by the local destruction of the optic nerve, brain stem, basal ganglia, and spinal cord, and certain areas of white matter in the brain. Triggers for this pathology are linked to immune system activity or failure of the myelin producing cells. MS can be classified as an inflammatory disease, but in addition, a dysregulation of the extrinsic pathways of coagulation has been repeatedly indicated (37–40). Excitingly, a recent publication by Göbel et al. suggests that the contact system and FXII in particular, might be a directly involved in the onset of MS (41). It was demonstrated that when an inflammatory response against the CNS is evoked in a mouse model for MS, FXII-knockout mice show a delayed disease onset and reduced disease severity compared to their wild-type counter parts (WT). The role of FXII in this context is critically dependent on the expression of the cell-surface receptor CD87 by dendritic cells, which is crucial for T-cell differentiation. Interestingly, CD87 is also known as uPAR, a key receptor in the urokinase plasminogen activation system, that mediates plasminogen activation on endothelial cells but also has cell signaling properties during FXII binding that influence angiogenesis (42). These recent studies indicate a novel role for FXII in MS, which is independent of intrinsic coagulation or the kallikrein–kinin system. However, a contribution of bradykinin or its metabolites to MS is still probable: blockade of the kinin B1 receptor, which is mainly expressed at sites of inflammation, reduces pathology in mouse models for MS by preventing T-cell migration into the nervous tissue by restoring excessive permeability of the blood–brain barrier (43). These combined studies point out that the role of FXII in immune modulation and inflammation in extravascular tissues may so far have been underappreciated.

## Two External Enzymes That Feed into the Contact System During Inflammation

It can be assumed that the mechanisms that drive physiological contact system activation are restricted to the same factors that are required for surface-triggered FXII-dependent coagulation (or pro-pKal activation) *in vitro*. In this context, pKal is the main enzyme that cleaves and activates FXII. However, extensive studies on endothelial cells have identified new external players that feed into the contact system, both by acting as pro-pKal activators (44, 45). In this next section, we will review examples of two enzymes from the serine-protease family that can feed into the contact system.

### Plasmin

Plasmin is foremost known for its thrombolytic function through fibrin breakdown and has strong therapeutic value in the treatment of thrombotic pathology (e.g., stroke). However, plasminogen activation can take place in the complete absence of a thrombus on the surface of endothelial cells in a receptor-mediated manner by the urokinase plasminogen activation system. Hypoxia is one of the triggers for expression of the required receptors (46). This among others helps to explain the generation of fibrinolytic activity during attacks of (fibrin-poor) thrombotic microangiopathy (47).

Hereditary angioedema is characterized by swelling attacks of the extremities, face, trunk, airway, or viscera of the abdomen. The onset can be spontaneous or secondary to trauma. The contact system and bradykinin production are heavily implicated, as HAE is often related to C1inh deficiency (48), as well as by mutations in FXII (49), and can be treated with kinin B2 receptor antagonists, C1inh reconstitution therapy, and antagonists of pKal (50). The mechanisms that underlie the attacks are currently unknown, but strikingly similar symptoms are seen in a subset of stroke patients (~5%) who undergo thrombolytic therapy (51). Furthermore, thrombolytic therapy after stroke exacerbates brain edema (52) with kallikrein–kinin system as known mediator (53). This connects well to the finding that the contact system is systemically activated after administration of plasminogen activators and the capacity of plasmin to cleave and activate FXII in a mechanism that closely resembles the function of pKal (28, 54). Another noteworthy influence of plasmin on the contact system is that it has the potential to “prime” HK for kallikrein-mediated liberation of bradykinin (55). We recently reported that three types of FXII mutations that cause HAE enhance the capacity of plasmin to cleave and activate FXII through introduction of novel enzymatic cleavage sites, leading to uncontrolled bradykinin production despite the presence of normal C1inh levels (29). Interestingly, the plasminogen activation system is linked in many of the inflammatory conditions described above, ranging from bacterial plasminogen activators to concurrent activation of plasminogen and the contact system in anaphylaxis (27, 30, 32). Based on these combined findings, we propose that plasmin is of importance for FXII-mediated bradykinin production in a context that expands beyond HAE.

### Elastase

Several cell types of the (innate) immune system, such as mast cells, basophils, and neutrophils contain and release elastase. This enzyme has been extensively studied as an inflammatory mediator in lung injury. Elastase has the capacity to cleave FXII into 28 kDa and 52 kDa fragments in peripheral blood. This destroys its procoagulant activity through removal of the surface-binding domains (56, 57), but does not directly activate the FXII molecule. As a result, elastase is currently seen as a negative regulator of the plasma contact system, but is this really the case? The cleavage pattern that FXII generates in response to elastase is strikingly similar to that of pKal, indicating that the cleavage sites for elastase and pKal in FXII are in close vicinity to each other. At this point, it is noteworthy to remember that pKal exerts a similar function during “classic” contact activation, removing the surface-binding domains of FXII to yield a fragmented active form of FXII with selective pro-pKal activating properties. In case of elastase cleavage, the soluble fragment has not (yet) been activated. In a mirroring mechanism, elastase is able cleave the light chain of HK. This eliminates its procoagulant properties, but leaves the kinin sequence untouched (56). It is attractive to hypothesize that this “primes” FXII for activation by pKal (or plasmin) and HK for liberation of bradykinin by pKal in the fluid phase. Finally, elastase can proteolytically inactivate α_2_-antiplasmin and C1inh (58). Taken together, the combined properties seem to point at the potential of elastase as a positive regulator of contact system activation, while shifting its actions toward a pro-inflammatory focus.

## Cleavage Does Not Equal Destruction: A Conceptual Model for Two-Stage Fluid Phase Activation of Factor XII

### “Classic” Contact Activation

As discussed earlier, the pattern by which FXII is cleaved by pKal is decisive in determining whether it acts as a procoagulant or pro-inflammatory enzyme (11) (Figure 1, left column). Three pKal-sensitive cleavage sites on FXII have been identified: R334, R343, and R353 [mature protein numbering (59)]. In the “classic” model of contact activation, α-FXII_A_ is formed first by cleavage at R353, which “locks” this molecule into a two-chain active conformation. In short, R353 cleavage is critical for FXII activation (59). Next, R334 cleavage generates β-FXII_A_, a fluid phase pro-pKal activator. The functional consequences of R343 cleavage are still unknown. However, since pKal is not the only serine protease that is able to cleave FXII, the sequence of events that occur during physiological FXII activation may be different than were originally discovered during surface-triggered contact system activation *in vitro*.

### A “Priming” Model for FXII-HAE

We recently reported that mutations in FXII that cause HAE (FXII-HAE) introduce new cleavage sites in the unstructured proline-rich region, which accelerate fluid phase activation by plasmin (29) (Figure 1, middle column). These sites are near to the R334 site, which pKal usually cleaves to generate β-FXII_A_ after initial activation. Our findings in FXII-HAE have led us to hypothesize that during activation of FXII-HAE mutants, the unactivated FXII protein is fragmented in solution *first*, rather than *after* initial activation. This step not only eliminates its procoagulant properties but also removes a functional sequence that shields site R353. This “primes” the FXII molecule and lowers the threshold for fluid phase activation by pKal or plasmin.

### Factor XII “Priming” by Elastase

Based on available biochemical data, the currently unidentified cleavage site for elastase is in the very same region as the FXII-HAE mutations, as well as near R334 (Figure 1, right column). In analogy to the mechanism described above, we propose that when elastase cleaves FXII, it converts the molecule into a fragment that is unable to generate clotting activity, but is has a significantly increased propensity for activation by pKal or plasmin in the fluid phase.

## Summary

**Figure 2:**
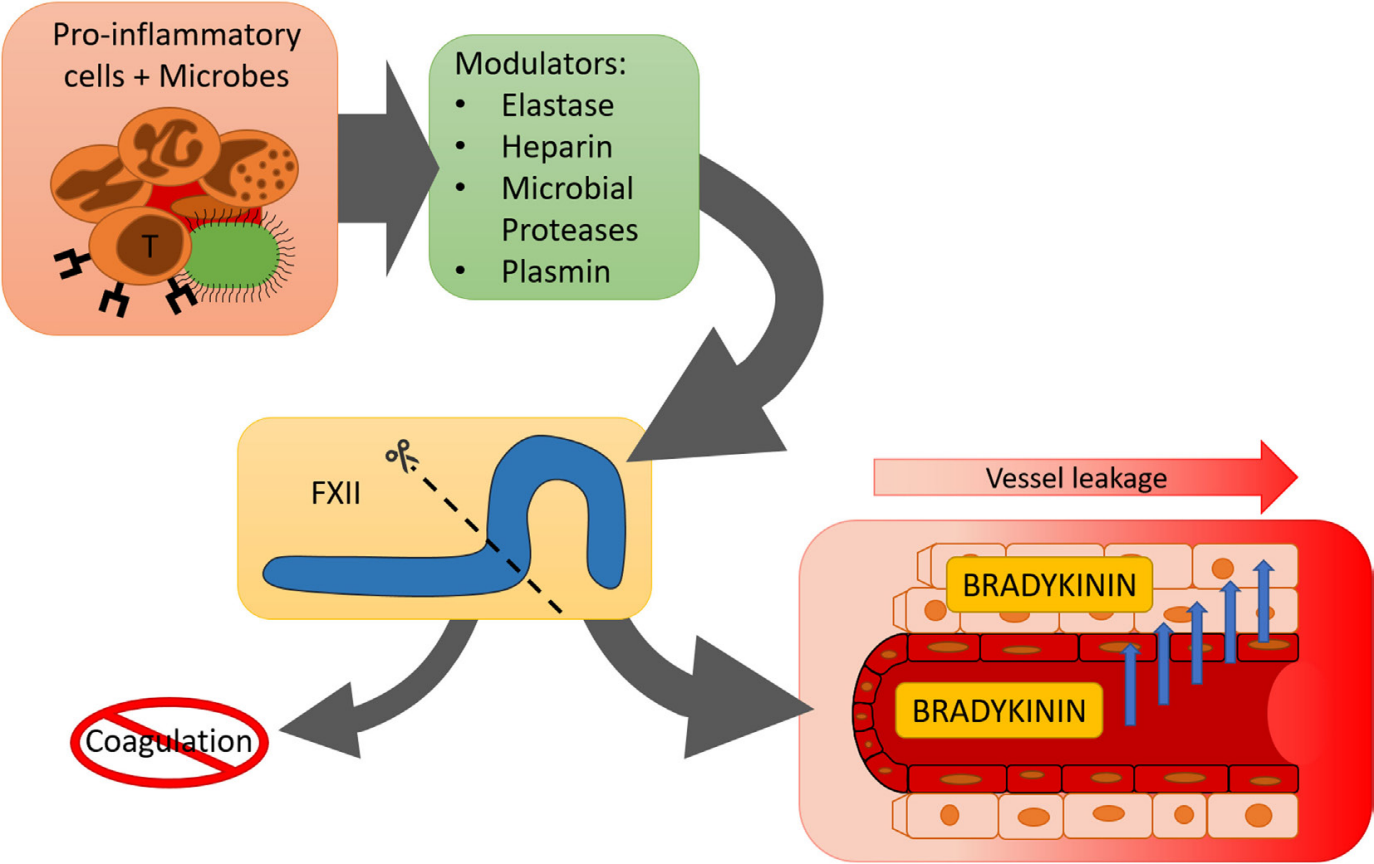
**Schematic overview of various pro-inflammatory modulators of the contact system**.

Accumulating evidence shows that the contact system is involved in inflammatory mechanisms that are not directly linked to blood coagulation (Figure 2). Alternative enzymatic processing of FXII by non-contact system enzymes may help to explain how the contact system “chooses” its direction.

## Author Contributions

BNJ, SM, and CM performed literature searches and wrote the manuscript.

## Conflict of Interest Statement

The authors declare that the research was conducted in the absence of any commercial or financial relationships that could be construed as a potential conflict of interest.
